# Predicting the effect of missense mutations on protein function: analysis with Bayesian networks

**DOI:** 10.1186/1471-2105-7-405

**Published:** 2006-09-06

**Authors:** Chris J Needham, James R Bradford, Andrew J Bulpitt, Matthew A Care, David R Westhead

**Affiliations:** 1 School of Computing, University of Leeds, Leeds, LS2 9JT, UK; 2 Institute of Molecular and Cellular Biology, University of Leeds, Leeds, LS2 9JT, UK

## Abstract

**Background:**

A number of methods that use both protein structural and evolutionary information are available to predict the functional consequences of missense mutations. However, many of these methods break down if either one of the two types of data are missing. Furthermore, there is a lack of rigorous assessment of how important the different factors are to prediction.

**Results:**

Here we use Bayesian networks to predict whether or not a missense mutation will affect the function of the protein. Bayesian networks provide a concise representation for inferring models from data, and are known to generalise well to new data. More importantly, they can handle the noisy, incomplete and uncertain nature of biological data. Our Bayesian network achieved comparable performance with previous machine learning methods. The predictive performance of learned model structures was no better than a naïve Bayes classifier. However, analysis of the posterior distribution of model structures allows biologically meaningful interpretation of relationships between the input variables.

**Conclusion:**

The ability of the Bayesian network to make predictions when only structural or evolutionary data was observed allowed us to conclude that structural information is a significantly better predictor of the functional consequences of a missense mutation than evolutionary information, for the dataset used. Analysis of the posterior distribution of model structures revealed that the top three strongest connections with the class node all involved structural nodes. With this in mind, we derived a simplified Bayesian network that used just these three structural descriptors, with comparable performance to that of an all node network.

## Background

An important aspect of the post-genomic era is to understand the biological effects of inherited variations between individuals. For instance, a key problem for the pharmaceutical industry is to understand variations in drug treatment responses among individuals at the molecular level. A single nucleotide polymorphism (SNP) is a mutation, such as an insertion, deletion or substitution, observed in the genomic DNA of individuals of the same species. When the SNP results in an amino acid substitution in the protein product of the gene, it is called a missense mutation. A missense mutation can have various phenotypic effects although we restrict ourselves here to the simplified task of predicting whether a missense mutation has an effect or no effect on protein function.

The wealth of SNP data now available [1-4] has prompted a number of studies on the functional consequences of SNPs. For example, Wang and Moult [5] and Ramensky *et al*. [6] showed that most of the detrimental missense mutations affect protein function indirectly through effects on protein structural stability particularly disruption to the protein hydrophobic core. The evolutionary properties of the mutated residue may also be important determinants of its effect on protein function [7-9], since conserved amino acids tend to be functionally important or critical in maintaining structural integrity. A number of groups have developed strategies to predict the effects of missense mutations by using structural or evolutionary information, or a combination of both. Most of these methods claim prediction accuracies of between 70 – 80% although comparison is extremely difficult due to the use of different data sets and criteria for assigning a mutation as having an effect or not. Chasman and Adams [7] proposed a probabilistic method, and Krishnan and Westhead [10] evaluated decision trees and support vector machines. Herrgard *et al*. [11] used structural motifs called Fuzzy Functional Forms to predict the effects of amino acid mutations on enzyme catalytic activity. Deleterious human alleles were predicted by Sunyaev *et al*. [12] using mostly structural information. By contrast, [13] used purely sequence homology data in their SIFT (Sorting Intolerant From Tolerant) algorithm, although adding structural information resulted in significant improvements [14]. Subsequent work has compared SIFT to SVMs and random forests [15]. Cai *et al*. [16] used a Bayesian framework to predict pathogenic SNPs. Verzilli *et al*. [17] applied a hierarchical Bayesian multivariate adaptive regression spline (hierarchical BMARS) model for binary classification of the functional consequences of SNPs. Within this model, samples from the posterior distribution were used to highlight properties of the mutated residue that are most important in predicting its effect on protein function.

All these methods require either structural or evolutionary data to be available for predictions to be possible. However, there are many proteins that lack any detectable sequence homology to known proteins or a solved 3D structure. In these cases, many prediction methods break down. Therefore a method is needed that can combine both structural and evolutionary information but at the same time tolerate the absence of either without manual intervention. With this in mind we have applied Bayesian networks to the problem of predicting the consequences of a missense mutation on protein function. Bayesian networks are probabilistic graphical models which provide a neat compact representation for expressing joint probability distributions and inference. The representation and use of probability theory makes Bayesian networks suitable for learning from incomplete datasets, expressing causal relationships, combining domain knowledge and data, and avoiding over-fitting a model to training data. As such, a host of applications in computational biology (for example, see [18-20]) have used Bayesian networks and Bayesian learning methodologies [21-23]. Our detailed evaluation of Bayesian network performance in this work is likely to be valuable to many groups working with Bayesian networks and biological data.

### Bayesian networks

Our recent primer [24] introduces Bayesian networks to the computational biologist. Briefly, given a set of variables **x **= {*x*_1_,..., *x*_*N*_}, which are represented as nodes in the Bayesian network, a set of directed edges representing relationships between nodes can be defined in a graph structure. To allow efficient inference and learning, a directed acyclic graph (DAG) must be formed, which exploits the conditional independence relations between variables. Using this model structure, model parameters *θ *in the form of conditional probability distributions (CPDs) between the connected variables may be learned. With discrete data, these model parameters take the form of conditional probability tables (CPTs). Throughout this work, we have used the Bayes Net Toolbox for MATLAB (BNT) [25]. The code used to produce the results presented in this paper is available on request from the authors.

#### Learning from complete data

The Bayesian learning paradigm can be summarised as:

*p*(**x**|*D*) = ∫*p*(**x**|*θ*)*p*(*θ*|*D*)*dθ*

I.e., the predictive distribution for a new example observation, given a set of training examples *D *can be calculated by averaging over all possible models *θ *the likelihood of the example **x **given the model, multiplied by the likelihood of the model given the training data. For a given model structure S
 MathType@MTEF@5@5@+=feaafiart1ev1aaatCvAUfKttLearuWrP9MDH5MBPbIqV92AaeXatLxBI9gBamrtHrhAL1wy0L2yHvtyaeHbnfgDOvwBHrxAJfwnaebbnrfifHhDYfgasaacH8akY=wiFfYdH8Gipec8Eeeu0xXdbba9frFj0=OqFfea0dXdd9vqai=hGuQ8kuc9pgc9s8qqaq=dirpe0xb9q8qiLsFr0=vr0=vr0dc8meaabaqaciaacaGaaeqabaWaaeGaeaaakeaaimaacqWFse=uaaa@3845@ the model *θ *can be thought of as the model parameters that encode the conditional probability distributions between variables and their parents in S
 MathType@MTEF@5@5@+=feaafiart1ev1aaatCvAUfKttLearuWrP9MDH5MBPbIqV92AaeXatLxBI9gBamrtHrhAL1wy0L2yHvtyaeHbnfgDOvwBHrxAJfwnaebbnrfifHhDYfgasaacH8akY=wiFfYdH8Gipec8Eeeu0xXdbba9frFj0=OqFfea0dXdd9vqai=hGuQ8kuc9pgc9s8qqaq=dirpe0xb9q8qiLsFr0=vr0=vr0dc8meaabaqaciaacaGaaeqabaWaaeGaeaaakeaaimaacqWFse=uaaa@3845@.

#### Learning from incomplete data

One advantage of using Bayesian networks is that it is possible to learn model parameters from incomplete training data i.e. in cases where variables are missing. To learn from incomplete data, we used the Expectation-Maximisation (EM) algorithm, which estimates missing values by computing the expected values and updating parameters using these expected values as if they were observed values.

#### Structure learning

A fully connected network structure captures relationships (dependencies) between all of the variables. A simpler, more compact model may be produced if conditional independencies between variables are learned. To do this, we used the greedy search algorithm from the Matlab-based structure learning package (SLP) [26] with tabular CPDs and uninformative Dirichlet priors (BDeu). The greedy search algorithm starts with a graph with no edges between the nodes, and aims to maximise a score function: either the full Bayesian posterior or the Bayesian Information Criterion (BIC). At each stage, the neighbourhood of the current graph (the set of graphs that differ by adding, reversing or deleting an edge) are considered, and the one with the highest score is chosen, until convergence. We use the notation of Heckerman, where S
 MathType@MTEF@5@5@+=feaafiart1ev1aaatCvAUfKttLearuWrP9MDH5MBPbIqV92AaeXatLxBI9gBamrtHrhAL1wy0L2yHvtyaeHbnfgDOvwBHrxAJfwnaebbnrfifHhDYfgasaacH8akY=wiFfYdH8Gipec8Eeeu0xXdbba9frFj0=OqFfea0dXdd9vqai=hGuQ8kuc9pgc9s8qqaq=dirpe0xb9q8qiLsFr0=vr0=vr0dc8meaabaqaciaacaGaaeqabaWaaeGaeaaakeaaimaacqWFse=uaaa@3845@^*h *^is a model structure hypothesis. From Bayes' theorem the posterior distribution for network structures *p*(S
 MathType@MTEF@5@5@+=feaafiart1ev1aaatCvAUfKttLearuWrP9MDH5MBPbIqV92AaeXatLxBI9gBamrtHrhAL1wy0L2yHvtyaeHbnfgDOvwBHrxAJfwnaebbnrfifHhDYfgasaacH8akY=wiFfYdH8Gipec8Eeeu0xXdbba9frFj0=OqFfea0dXdd9vqai=hGuQ8kuc9pgc9s8qqaq=dirpe0xb9q8qiLsFr0=vr0=vr0dc8meaabaqaciaacaGaaeqabaWaaeGaeaaakeaaimaacqWFse=uaaa@3845@^*h*^|*D*) is proportional to the marginal likelihood of the data *p*(*D*|S
 MathType@MTEF@5@5@+=feaafiart1ev1aaatCvAUfKttLearuWrP9MDH5MBPbIqV92AaeXatLxBI9gBamrtHrhAL1wy0L2yHvtyaeHbnfgDOvwBHrxAJfwnaebbnrfifHhDYfgasaacH8akY=wiFfYdH8Gipec8Eeeu0xXdbba9frFj0=OqFfea0dXdd9vqai=hGuQ8kuc9pgc9s8qqaq=dirpe0xb9q8qiLsFr0=vr0=vr0dc8meaabaqaciaacaGaaeqabaWaaeGaeaaakeaaimaacqWFse=uaaa@3845@^*h*^). The full Bayesian posterior can be calculated [[27], equation 35], or the BIC approximation can be used, which contains a term to describe how well the maximum likelihood model θ^
 MathType@MTEF@5@5@+=feaafiart1ev1aaatCvAUfKttLearuWrP9MDH5MBPbIqV92AaeXatLxBI9gBaebbnrfifHhDYfgasaacH8akY=wiFfYdH8Gipec8Eeeu0xXdbba9frFj0=OqFfea0dXdd9vqai=hGuQ8kuc9pgc9s8qqaq=dirpe0xb9q8qiLsFr0=vr0=vr0dc8meaabaqaciaacaGaaeqabaqabeGadaaakeaaiiGacuWF4oqCgaqcaaaa@2E79@_*s *_for structure S
 MathType@MTEF@5@5@+=feaafiart1ev1aaatCvAUfKttLearuWrP9MDH5MBPbIqV92AaeXatLxBI9gBamrtHrhAL1wy0L2yHvtyaeHbnfgDOvwBHrxAJfwnaebbnrfifHhDYfgasaacH8akY=wiFfYdH8Gipec8Eeeu0xXdbba9frFj0=OqFfea0dXdd9vqai=hGuQ8kuc9pgc9s8qqaq=dirpe0xb9q8qiLsFr0=vr0=vr0dc8meaabaqaciaacaGaaeqabaWaaeGaeaaakeaaimaacqWFse=uaaa@3845@^*h *^predicts the data *D*, and a term that punishes model complexity. For a model with *d *parameters, built from *N *samples, the BIC score is:

ln⁡p(D|Sh)≈ln⁡p(D|θ^s,Sh)−d2ln⁡N
 MathType@MTEF@5@5@+=feaafiart1ev1aaatCvAUfKttLearuWrP9MDH5MBPbIqV92AaeXatLxBI9gBamrtHrhAL1wy0L2yHvtyaeHbnfgDOvwBHrxAJfwnaebbnrfifHhDYfgasaacH8akY=wiFfYdH8Gipec8Eeeu0xXdbba9frFj0=OqFfea0dXdd9vqai=hGuQ8kuc9pgc9s8qqaq=dirpe0xb9q8qiLsFr0=vr0=vr0dc8meaabaqaciaacaGaaeqabaWaaeGaeaaakeaacyGGSbaBcqGGUbGBcqWGWbaCcqGGOaakcqWGebarcqGG8baFimaacqWFse=udaahaaWcbeqaaiabdIgaObaakiabcMcaPiabgIKi7kGbcYgaSjabc6gaUjabdchaWjabcIcaOiabdseaejabcYha8HGaciqb+H7aXzaajaWaaSbaaSqaaiabdohaZbqabaGccqGGSaalcqWFse=udaahaaWcbeqaaiabdIgaObaakiabcMcaPiabgkHiTmaalaaabaGaemizaqgabaGaeGOmaidaaiGbcYgaSjabc6gaUjabd6eaobaa@5B51@

#### Inference with missing data

**Figure 1:**
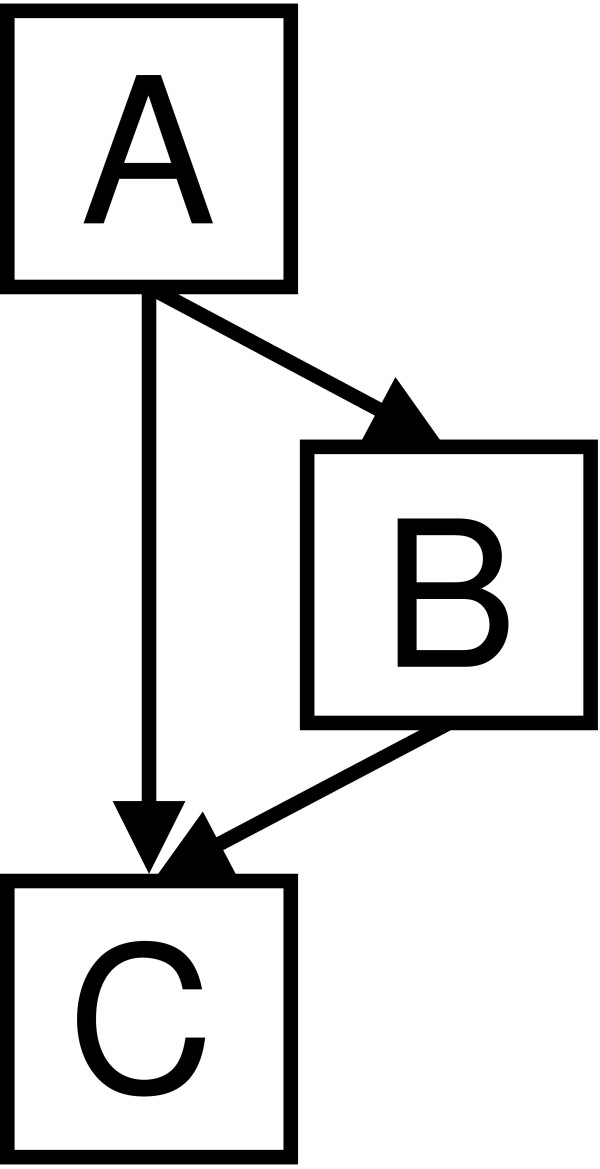
**Example 3 node Bayesian network**. Example 3 node Bayesian network.

Knowledge of the conditional probability distributions between variables allows us to make predictions about the expected states of variables even if some variables are missing from the test data. For example, if structural information about a test missense mutation is not available, we can still infer whether the mutation has a functional effect on the protein or not by marginalising over the unknown variables. This is illustrated in a very simple Bayesian network with three nodes, A, B, C, which can take the values {*a*_1_,..., aNA
 MathType@MTEF@5@5@+=feaafiart1ev1aaatCvAUfKttLearuWrP9MDH5MBPbIqV92AaeXatLxBI9gBaebbnrfifHhDYfgasaacH8akY=wiFfYdH8Gipec8Eeeu0xXdbba9frFj0=OqFfea0dXdd9vqai=hGuQ8kuc9pgc9s8qqaq=dirpe0xb9q8qiLsFr0=vr0=vr0dc8meaabaqaciaacaGaaeqabaqabeGadaaakeaacqWGHbqydaWgaaWcbaGaemOta40aaSbaaWqaaiabdgeabbqabaaaleqaaaaa@308B@}, {*b*_1_,..., bNB
 MathType@MTEF@5@5@+=feaafiart1ev1aaatCvAUfKttLearuWrP9MDH5MBPbIqV92AaeXatLxBI9gBaebbnrfifHhDYfgasaacH8akY=wiFfYdH8Gipec8Eeeu0xXdbba9frFj0=OqFfea0dXdd9vqai=hGuQ8kuc9pgc9s8qqaq=dirpe0xb9q8qiLsFr0=vr0=vr0dc8meaabaqaciaacaGaaeqabaqabeGadaaakeaacqWGIbGydaWgaaWcbaGaemOta40aaSbaaWqaaiabdkeacbqabaaaleqaaaaa@308F@}, and {*c*_1_,..., cNC
 MathType@MTEF@5@5@+=feaafiart1ev1aaatCvAUfKttLearuWrP9MDH5MBPbIqV92AaeXatLxBI9gBaebbnrfifHhDYfgasaacH8akY=wiFfYdH8Gipec8Eeeu0xXdbba9frFj0=OqFfea0dXdd9vqai=hGuQ8kuc9pgc9s8qqaq=dirpe0xb9q8qiLsFr0=vr0=vr0dc8meaabaqaciaacaGaaeqabaqabeGadaaakeaacqWGJbWydaWgaaWcbaGaemOta40aaSbaaWqaaiabdoeadbqabaaaleqaaaaa@3093@} respectively and a structure given by Figure 1. The joint probability over all the variables is:

*p*(*A*, *B*, *C*) = *p*(*A*)*p*(*B*|*A*)*p*(*C*|*A*, *B*)

Each of the probabilities can be expressed as a conditional probability table in this discrete case. If we wish to infer the value of *C *given *A *= *a*_*i *_and *B *= *b*_*j *_then we can calculate the probability of *C *taking each of the possible values, *C *= *c*_*k *_for *k *= 1,..., *N*_*C *_by *p*(*c*_*k*_|*a*_*i*_, *b*_*j*_) read from CPTs. If we wish to infer the value of C given only the value of A, we can marginalise over the unknown variables (in this case, B). Thus:

p(ck|ai)=∑bj∈Bp(bj|ai)p(ck|ai,bj)
 MathType@MTEF@5@5@+=feaafiart1ev1aaatCvAUfKttLearuWrP9MDH5MBPbIqV92AaeXatLxBI9gBaebbnrfifHhDYfgasaacH8akY=wiFfYdH8Gipec8Eeeu0xXdbba9frFj0=OqFfea0dXdd9vqai=hGuQ8kuc9pgc9s8qqaq=dirpe0xb9q8qiLsFr0=vr0=vr0dc8meaabaqaciaacaGaaeqabaqabeGadaaakeaacqWGWbaCcqGGOaakcqWGJbWydaWgaaWcbaGaem4AaSgabeaakiabcYha8jabdggaHnaaBaaaleaacqWGPbqAaeqaaOGaeiykaKIaeyypa0ZaaabuaeaacqWGWbaCcqGGOaakcqWGIbGydaWgaaWcbaGaemOAaOgabeaakiabcYha8jabdggaHnaaBaaaleaacqWGPbqAaeqaaOGaeiykaKIaemiCaaNaeiikaGIaem4yam2aaSbaaSqaaiabdUgaRbqabaGccqGG8baFcqWGHbqydaWgaaWcbaGaemyAaKgabeaakiabcYcaSiabdkgaInaaBaaaleaacqWGQbGAaeqaaOGaeiykaKcaleaacqWGIbGydaWgaaadbaGaemOAaOgabeaaliabgIGiolabdkeacbqab0GaeyyeIuoaaaa@5815@

## Results and discussion

The systematic unbiased mutagenesis dataset of lac repressor [28,29] and T4 lysozyme [30,31] were used to train and validate the Bayesian networks. Classification of 'effect' and 'no effect' mutations was based on that of [17] in which only those mutations resulting in a significant loss of function were considered 'effect' mutations. As a result, our lac repressor dataset consisted of 823 effect and 2422 no effect mutations, and our T4 lysozyme dataset contained 312 effect and 1320 no effect mutations.

A total of fourteen variables were used to predict whether or not a missense mutation affects protein function (Table 1; Note also the abbreviations introduced – taken from the dataset of [17]). All these variables have been implicated in previous studies as useful in discriminating 'effect' from 'no effect' mutations. Six of the variables are continuous (*ac*, *rac*, *rent*, *nrent*, *bf*, and *nbf*), the rest are discrete binary. The variables (excluding the class node) can also be sorted into three groups based on the type of biological information they give: structural, evolutionary, or in the case of *nrent* structural and evolutionary information.

**Table 1:** Attributes used for predicting functional effects of missense mutations

**Abbreviation**	**Type**	**Description**	**Information**
*effect*	Discrete	Effect of mutation on functionality	Class

*ac*	Continuous	Solvent accessible area of native AA	
*rac*	Continuous	Accessibility relative to maximum accessibility in training set	
*bf*	Continuous	Normalised B-factor of native AA	
*nbf*	Continuous	Normalised B-factor of structural neighbourhood of native AA	Structural
*bur*	Discrete	Mutant AA is charged AA at buried site	
*trn*	Discrete	Mutant AA occurs at glycine or proline in a turn	
*hlx*	Discrete	Mutant AA occurs in helical region and involves glycine or proline	
*ifc*	Discrete	Native AA is near subunit interface	

*nrent*	Continuous	Phylogenetic entropy of structural neighbourhood of native AA	Structural + Evolutionary

*rent*	Continuous	Normalised phylogenetic entropy of native AA	
*cnsd*	Discrete	Native AA is at conserved position in phylogenetic profile	
*ncnsd*	Discrete	Native AA is near conserved position in phylogenetic profile	Evolutionary
*uslaa*	Discrete	Mutant AA is not in phylogenetic profile	
*uslby*	Discrete	Mutant AA is not in the smallest AA class that includes the phylogenetic profile	

We used two basic types of Bayesian network structure in this study: naïve and learned. In the naïve structure, the *effec*t node is a parent to all the other nodes in the network structure. Details of the learned structure are provided later. On each of these structures we performed seven experiments:

• *all*:*all*: 15 node network trained and tested using all 14 variables listed in Table 1.

• *all*: *noS*: 15 node network trained on all variables, tested with evolutionary information only (*ac*, *rac*, *bf*, *nbf*, *bur*, *trn*, *hlx*, *ifc*, *nrent* nodes hidden).

• *noS*:*noS*: 6 node network (structural nodes missing) trained and tested with evolutionary information only.

• *all*:*noE*: 15 node network trained on all variables, tested with structural information only (*nrent*, *rent*, *cnsd*, *ncnsd*, *uslaa*, *uslby* nodes hidden).

• *noE*:*noE*: 9 node network (evolutionary nodes missing) trained and tested with structural information only.

• *all*:*key*: 15 node network trained on all variables, tested using three key variables (*ac*, *bur*, *bf*). These key variables were identified by analysing a number of learned structures.

• *key*:*key*: 4 node network trained and tested using key variables only.

Results of these experiments are presented in Tables 2 and 3. We carried out both homogeneous and heterogeneous cross-validation tests. Homogeneous cross-validation was performed on both lysozyme and lac repressor datasets separately, and a mixed set in which the two datasets were pooled. In each case, data were randomised and divided into 10 equal parts. One part was used as the test set and the remainder as the training set. This procedure was repeated 10 times so that each example (here it is each mutation) was used exactly once for testing. The mean and standard deviation of the ten results were then calculated. In heterogeneous cross-validation, the data set of one protein (e.g. lac repressor) was used as the training set and that of the other protein (e.g. lysozyme) was used as the test set.

**Table 2:** Results with a naïve Bayes classifier.

Cross-validation	Trained on:	All	All	NoS	All	NoE	All	key
	Tested on:	All	NoS	NoS	NoE	NoE	key	key
mixed	AUC	0.83 ± 0.01	0.70 ± 0.02	0.70 ± 0.02	0.81 ± 0.02	0.81 ± 0.02	0.80 ± 0.02	0.79 ± 0.01
	MCC	0.44 ± 0.04	0.27 ± 0.03	0.27 ± 0.03	0.43 ± 0.03	0.43 ± 0.03	0.41 ± 0.02	0.35 ± 0.06
	Overall error rate	0.19 ± 0.01	0.24 ± 0.01	0.24 ± 0.01	0.18 ± 0.01	0.18 ± 0.01	0.18 ± 0.01	0.21 ± 0.00
	Effect error rate	0.35 ± 0.05	0.52 ± 0.03	0.52 ± 0.03	0.26 ± 0.07	0.26 ± 0.07	0.24 ± 0.07	0.41 ± 0.04
	No effect error rate	0.15 ± 0.02	0.18 ± 0.01	0.18 ± 0.01	0.17 ± 0.01	0.17 ± 0.01	0.17 ± 0.02	0.17 ± 0.03
	sensitivity	0.47 ± 0.12	0.37 ± 0.06	0.37 ± 0.06	0.37 ± 0.06	0.37 ± 0.06	0.36 ± 0.09	0.38 ± 0.16
	specificity	0.92 ± 0.03	0.88 ± 0.02	0.88 ± 0.02	0.96 ± 0.02	0.96 ± 0.02	0.96 ± 0.03	0.92 ± 0.05

lac rep	AUC	0.84 ± 0.02	0.74 ± 0.02	0.74 ± 0.02	0.82 ± 0.02	0.82 ± 0.02	0.80 ± 0.02	0.80 ± 0.02
	MCC	0.47 ± 0.03	0.33 ± 0.06	0.33 ± 0.06	0.46 ± 0.04	0.46 ± 0.04	0.44 ± 0.03	0.39 ± 0.05
	Overall error rate	0.18 ± 0.01	0.23 ± 0.01	0.23 ± 0.01	0.18 ± 0.01	0.18 ± 0.01	0.19 ± 0.01	0.21 ± 0.00
	Effect error rate	0.27 ± 0.05	0.40 ± 0.04	0.40 ± 0.04	0.20 ± 0.06	0.20 ± 0.06	0.18 ± 0.09	0.36 ± 0.05
	No effect error rate	0.16 ± 0.02	0.19 ± 0.02	0.19 ± 0.02	0.18 ± 0.02	0.18 ± 0.02	0.19 ± 0.03	0.18 ± 0.03
	sensitivity	0.47 ± 0.10	0.36 ± 0.12	0.36 ± 0.12	0.37 ± 0.08	0.38 ± 0.08	0.34 ± 0.12	0.41 ± 0.13
	specificity	0.93 ± 0.03	0.92 ± 0.04	0.92 ± 0.04	0.96 ± 0.02	0.96 ± 0.02	0.97 ± 0.04	0.92 ± 0.04

lysozyme	AUC	0.83 ± 0.02	0.68 ± 0.04	0.68 ± 0.05	0.81 ± 0.04	0.81 ± 0.04	0.78 ± 0.04	0.77 ± 0.04
	MCC	0.40 ± 0.05	0.23 ± 0.06	0.23 ± 0.06	0.38 ± 0.08	0.38 ± 0.08	0.36 ± 0.11	0.28 ± 0.09
	Overall error rate	0.17 ± 0.02	0.24 ± 0.01	0.24 ± 0.02	0.17 ± 0.03	0.17 ± 0.03	0.16 ± 0.02	0.21 ± 0.03
	Effect error rate	0.40 ± 0.05	0.63 ± 0.05	0.63 ± 0.05	0.39 ± 0.12	0.39 ± 0.12	0.33 ± 0.13	0.54 ± 0.09
	No effect error rate	0.13 ± 0.02	0.15 ± 0.01	0.15 ± 0.01	0.13 ± 0.03	0.13 ± 0.03	0.15 ± 0.02	0.14 ± 0.02
	Sensitivity	0.43 ± 0.11	0.39 ± 0.07	0.39 ± 0.07	0.38 ± 0.17	0.38 ± 0.17	0.28 ± 0.09	0.36 ± 0.11
	Specificity	0.93 ± 0.03	0.84 ± 0.02	0.84 ± 0.02	0.93 ± 0.07	0.93 ± 0.07	0.97 ± 0.01	0.89 ± 0.04

Train: lac rep	AUC	0.80	0.66	0.67	0.78	0.78	0.77	0.77
	MCC	0.40	0.23	0.23	0.35	0.35	0.35	0.35
	Overall error rate	0.20	0.27	0.24	0.17	0.17	0.16	0.16
Test: lysozyme	Effect error rate	0.52	0.65	0.63	0.41	0.41	0.32	0.32
	No effect error rate	0.10	0.14	0.15	0.14	0.14	0.15	0.16
	Sensitivity	0.58	0.46	0.39	0.33	0.33	0.26	0.26
	Specificity	0.85	0.80	0.84	0.95	0.95	0.97	0.97

Train: lysozyme	AUC	0.81	0.71	0.71	0.80	0.80	0.79	0.79
	MCC	0.43	0.37	0.37	0.41	0.41	0.42	0.42
	Overall error rate	0.20	0.22	0.22	0.20	0.20	0.19	0.19
Test: lac rep	Effect error rate	0.34	0.43	0.43	0.25	0.25	0.18	0.18
	No effect error rate	0.17	0.17	0.17	0.19	0.19	0.20	0.20
	Sensitivity	0.45	0.46	0.46	0.33	0.33	0.30	0.30
	Specificity	0.92	0.88	0.88	0.96	0.96	0.98	0.98

Column: (1) trained on all variables, tested with all variables observed; (2) trained on all variables, tested without any structural information (NoS) – only evolutionary variables observed; (3) trained and tested using only five evolutionary nodes; (4) trained on all variables, tested without any evolutionary information (NoE) – only structural variables observed; (5) trained and tested using only eight structural nodes; (6) trained on all variables, tested with only key variables observed (see later section); (7) trained and tested using only the three key variables.

**Table 3:** Results with a learned Bayesian network.

Cross-validation	Trained on:	All	All	NoS	All	NoE	All	key
	Tested on:	All	NoS	NoS	NoE	NoE	key	key
mixed	AUC	0.84 ± 0.01	0.64 ± 0.01	0.70 ± 0.02	0.72 ± 0.02	0.82 ± 0.02	0.63 ± 0.03	0.80 ± 0.02
	MCC	0.46 ± 0.03	0.11 ± 0.03	0.10 ± 0.16	0.26 ± 0.22	0.44 ± 0.03	0.40 ± 0.04	0.40 ± 0.04
	Overall error rate	0.17 ± 0.01	0.67 ± 0.01	0.23 ± 0.00	0.36 ± 0.28	0.18 ± 0.01	0.18 ± 0.01	0.18 ± 0.01
	Effect error rate	0.27 ± 0.05	0.75 ± 0.01	0.15 ± 0.24	0.40 ± 0.25	0.29 ± 0.07	0.24 ± 0.06	0.25 ± 0.05
	No effect error rate	0.16 ± 0.01	0.11 ± 0.03	0.21 ± 0.03	0.29 ± 0.18	0.16 ± 0.02	0.18 ± 0.01	0.18 ± 0.01
	sensitivity	0.41 ± 0.07	0.93 ± 0.01	0.13 ± 0.21	0.51 ± 0.33	0.41 ± 0.08	0.31 ± 0.04	0.31 ± 0.09
	specificity	0.95 ± 0.02	0.15 ± 0.02	0.96 ± 0.07	0.68 ± 0.47	0.95 ± 0.03	0.97 ± 0.01	0.97 ± 0.01

lac rep	AUC	0.85 ± 0.01	0.47 ± 0.03	0.73 ± 0.02	0.70 ± 0.02	0.82 ± 0.02	0.61 ± 0.02	0.81 ± 0.02
	MCC	0.52 ± 0.02	0.11 ± 0.03	0.32 ± 0.04	0.43 ± 0.04	0.46 ± 0.05	0.42 ± 0.04	0.42 ± 0.03
	Overall error rate	0.17 ± 0.01	0.60 ± 0.01	0.24 ± 0.01	0.19 ± 0.01	0.18 ± 0.01	0.19 ± 0.01	0.19 ± 0.01
	Effect error rate	0.25 ± 0.03	0.72 ± 0.01	0.46 ± 0.03	0.20 ± 0.06	0.21 ± 0.05	0.17 ± 0.07	0.22 ± 0.06
	No effect error rate	0.15 ± 0.01	0.16 ± 0.02	0.19 ± 0.01	0.19 ± 0.01	0.18 ± 0.01	0.20 ± 0.01	0.19 ± 0.01
	sensitivity	0.51 ± 0.03	0.86 ± 0.02	0.40 ± 0.03	0.33 ± 0.03	0.38 ± 0.06	0.30 ± 0.02	0.33 ± 0.02
	specificity	0.94 ± 0.01	0.24 ± 0.02	0.88 ± 0.01	0.97 ± 0.01	0.96 ± 0.01	0.98 ± 0.01	0.97 ± 0.01

lysozyme	AUC	0.86 ± 0.02	0.51 ± 0.06	0.67 ± 0.05	0.78 ± 0.04	0.83 ± 0.05	0.70 ± 0.04	0.78 ± 0.05
	MCC	0.47 ± 0.06	0.09 ± 0.05	-	0.37 ± 0.10	0.40 ± 0.10	0.37 ± 0.12	0.34 ± 0.12
	Overall error rate	0.17 ± 0.03	0.75 ± 0.02	0.19 ± 0.00	0.16 ± 0.02	0.16 ± 0.02	0.16 ± 0.02	0.16 ± 0.02
	Effect error rate	0.38 ± 0.14	0.80 ± 0.01	-	0.30 ± 0.13	0.34 ± 0.11	0.32 ± 0.13	0.33 ± 0.14
	No effect error rate	0.10 ± 0.03	0.05 ± 0.08	0.19 ± 0.00	0.15 ± 0.02	0.14 ± 0.02	0.15 ± 0.02	0.15 ± 0.02
	Sensitivity	0.55 ± 0.19	0.98 ± 0.02	0.00 ± 0.00	0.29 ± 0.10	0.36 ± 0.09	0.30 ± 0.09	0.26 ± 0.09
	Specificity	0.90 ± 0.07	0.07 ± 0.02	1.00 ± 1.00	0.97 ± 0.02	0.95 ± 0.02	0.97 ± 0.01	0.97 ± 0.01

Train: lac rep	AUC	0.72	0.43	0.68	0.70	0.77	0.57	0.75
	MCC	0.30	-	0.23	0.21	0.36	0.34	0.35
	Overall error rate	0.17	0.19	0.27	0.21	0.17	0.17	0.17
Test: lysozyme	Effect error rate	0.33	-	0.65	0.57	0.41	0.35	0.35
	No effect error rate	0.16	0.19	0.14	0.16	0.14	0.15	0.15
	Sensitivity	0.20	0.00	0.46	0.25	0.35	0.26	0.26
	Specificity	0.98	1.00	0.80	0.92	0.94	0.97	0.97

Train: lysozyme	AUC	0.79	0.44	0.65	0.58	0.78	0.66	0.78
	MCC	0.41	-0.11	0.32	0.06	0.42	0.40	0.41
	Overall error rate	0.20	0.39	0.24	0.25	0.20	0.20	0.20
Test: lac rep	Effect error rate	0.22	0.84	0.46	0.30	0.26	0.23	0.23
	No effect error rate	0.19	0.28	0.19	0.25	0.19	0.20	0.19
	Sensitivity	0.32	0.13	0.40	0.01	0.35	0.30	0.33
	Specificity	0.97	0.78	0.88	1.00	0.96	0.97	0.97

See Table 2 for column details. Note that MCC score or effect rate cannot be shown if all mutations are predicted as 'no effect'.

### Naïve Bayes classifier

#### all:all

As expected, overall error rates of less than 20% were achieved in all cross validation tests with the *all*:*all *model (Table 2, column 1). These results are consistent with previous studies reporting accuracies of 70 – 80% on similar datasets using similar variables [7,10,17]. Furthermore, all AUC values (Area under ROC curve – see Evaluation measures in Methods section for details of all performance metrics), including those from heterogeneous cross validation were at least 0.80 indicating a robust classifier despite the naïvety of the network structure. We therefore used results on the *all*:*all *model as a benchmark for the six other experiments.

#### Missing structural information (all:noS and noS:noS)

Performance dropped significantly with a 6 node network utilising only evolutionary information (*noS*:*noS*, Table 2, Column 3), with most AUC values reduced by over 10% from the *all*:*all *model. In particular, with homogeneous cross validation on lysozyme data AUC value decreased from 0.83 to 0.68, and MCC value was as low as 0.23. Even when structural information was used in training the network (*all*:*noS*, Table 2, Column 2), results were not improved possibly because variables are treated as independent in a naïve structure and so variables with missing values have little influence when they are marginalised over.

#### Missing evolutionary information (all:noE and noE:noE)

In contrast to results achieved without structural information, there was little or no effect on performance when evolutionary information was either missing during testing (*all*:*noE*, Table 2, Column 4) or missing during both training and testing (*noE*:*noE*, Table 2, Column 5). Again, due to the naïvety of the structure, similar results were achieved by the *all*:*noE *and *noE*:*noE *models with AUC values of around 0.80 and overall error rates below 0.20.

Overall, results suggest that structural information is more important than evolutionary information in predicting the functional consequences of a missense mutation in both lac repressor and T4 lysozyme, for the dataset used. Indeed, although evolutionary information has some predictive power, utilising only structural information appears to be sufficient for accurate prediction, comparable to that of the *all*:*all *model.

#### A note on structural flexibility

It has previously been suggested that the B-factor and neighbourhood B-factor of the native amino acid are the most important predictors of functional effects of SNPs [17]. However, the need to use B-factor information limits a method to structures from X-ray crystallography; such information is not available for NMR structures (although these do have their own internal flexibility measures). We found that removing the *bf* and *nbf* nodes from the all node network made little significant difference to overall performance with AUCs ranging from 0.80 to 0.83 in homogeneous cross-validation and 0.78 and 0.82 in heterogeneous cross-validation (results not in Table). This suggests that accurate prediction is possible without using structural flexibility information, although that is not to say that structural flexibility is not important, rather, other variables have compensated effectively for its loss.

### Learned structure

**Figure 2:**
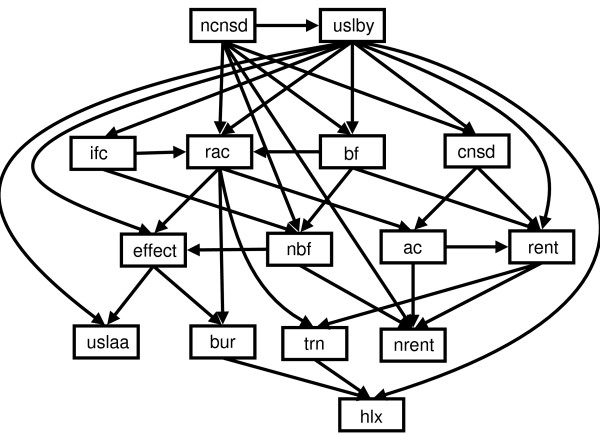
**Learned Bayesian network structure **S
 MathType@MTEF@5@5@+=feaafiart1ev1aaatCvAUfKttLearuWrP9MDH5MBPbIqV92AaeXatLxBI9gBamrtHrhAL1wy0L2yHvtyaeHbnfgDOvwBHrxAJfwnaebbnrfifHhDYfgasaacH8akY=wiFfYdH8Gipec8Eeeu0xXdbba9frFj0=OqFfea0dXdd9vqai=hGuQ8kuc9pgc9s8qqaq=dirpe0xb9q8qiLsFr0=vr0=vr0dc8meaabaqaciaacaGaaeqabaWaaeGaeaaakeaaimaacqWFse=uaaa@3845@. Learned Bayesian network structure S
 MathType@MTEF@5@5@+=feaafiart1ev1aaatCvAUfKttLearuWrP9MDH5MBPbIqV92AaeXatLxBI9gBamrtHrhAL1wy0L2yHvtyaeHbnfgDOvwBHrxAJfwnaebbnrfifHhDYfgasaacH8akY=wiFfYdH8Gipec8Eeeu0xXdbba9frFj0=OqFfea0dXdd9vqai=hGuQ8kuc9pgc9s8qqaq=dirpe0xb9q8qiLsFr0=vr0=vr0dc8meaabaqaciaacaGaaeqabaWaaeGaeaaakeaaimaacqWFse=uaaa@3845@ (using greedy search with BIC scoring function from mixed dataset). Key to node labels is shown in Table 1.

Using both the Bayesian and BIC scoring functions employed by the greedy search algorithm we learned structures from lac repressor and lysozyme datasets separately and the two datasets combined ('mixed'). As with the naïve Bayes classifier, we evaluated each structure using both homogeneous ten-fold and heterogeneous cross-validation. There was little significant difference in performance between the two scoring functions, or between structures learned on different datasets. The main difference was in the number of edges in the resulting DAGs. For our mixed dataset, there were 35 edges with BIC, and 48 with full Bayesian scoring. Using *Occam's Razor*, we prefer the simplest of equally good models, and take the Bayesian network structure learned from the mixed dataset, using the BIC scoring function, as our model structure S
 MathType@MTEF@5@5@+=feaafiart1ev1aaatCvAUfKttLearuWrP9MDH5MBPbIqV92AaeXatLxBI9gBamrtHrhAL1wy0L2yHvtyaeHbnfgDOvwBHrxAJfwnaebbnrfifHhDYfgasaacH8akY=wiFfYdH8Gipec8Eeeu0xXdbba9frFj0=OqFfea0dXdd9vqai=hGuQ8kuc9pgc9s8qqaq=dirpe0xb9q8qiLsFr0=vr0=vr0dc8meaabaqaciaacaGaaeqabaWaaeGaeaaakeaaimaacqWFse=uaaa@3845@, which is illustrated in Figure 2. With a harsher penalty for extra edges, the DAG learned using the BIC scoring function, should contain edges which are more likely to be biologically meaningful. It is important to note that the Bayesian networks with learned structure (or structure determined from conditional independence relations identified by an expert) capture the relationships between all the variables, and are not designed solely to discriminate for classification of a single variable based on the other variables. This is a significant advantage of the Bayesian network: we can infer the value of any variable(s) based on the value of any other variable, so we have constructed a model which can not only predict effect/no effect, but can infer the value of any of the attributes.

#### all:all

Little significant improvement in homogeneous cross validation performance was gained from using structure S
 MathType@MTEF@5@5@+=feaafiart1ev1aaatCvAUfKttLearuWrP9MDH5MBPbIqV92AaeXatLxBI9gBamrtHrhAL1wy0L2yHvtyaeHbnfgDOvwBHrxAJfwnaebbnrfifHhDYfgasaacH8akY=wiFfYdH8Gipec8Eeeu0xXdbba9frFj0=OqFfea0dXdd9vqai=hGuQ8kuc9pgc9s8qqaq=dirpe0xb9q8qiLsFr0=vr0=vr0dc8meaabaqaciaacaGaaeqabaWaaeGaeaaakeaaimaacqWFse=uaaa@3845@ (Table 3, column 1) over the simple naïve structure (Table 2, column 1). This was because the naïve structure is specifically designed for classification, whereas our learned structure is the 'best' structure for capturing the relationships between all of the variables. The learned structure S
 MathType@MTEF@5@5@+=feaafiart1ev1aaatCvAUfKttLearuWrP9MDH5MBPbIqV92AaeXatLxBI9gBamrtHrhAL1wy0L2yHvtyaeHbnfgDOvwBHrxAJfwnaebbnrfifHhDYfgasaacH8akY=wiFfYdH8Gipec8Eeeu0xXdbba9frFj0=OqFfea0dXdd9vqai=hGuQ8kuc9pgc9s8qqaq=dirpe0xb9q8qiLsFr0=vr0=vr0dc8meaabaqaciaacaGaaeqabaWaaeGaeaaakeaaimaacqWFse=uaaa@3845@ performs as well in classification of *effect* as the naïve structure, but has the added advantage that it can be used to predict the values of any of the variables, from any of the other variables.

**Figure 3:**
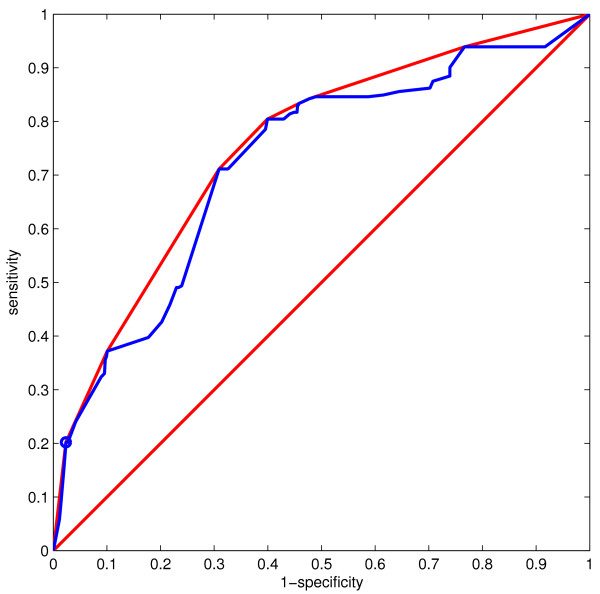
**ROC curve for learned structure **S
 MathType@MTEF@5@5@+=feaafiart1ev1aaatCvAUfKttLearuWrP9MDH5MBPbIqV92AaeXatLxBI9gBamrtHrhAL1wy0L2yHvtyaeHbnfgDOvwBHrxAJfwnaebbnrfifHhDYfgasaacH8akY=wiFfYdH8Gipec8Eeeu0xXdbba9frFj0=OqFfea0dXdd9vqai=hGuQ8kuc9pgc9s8qqaq=dirpe0xb9q8qiLsFr0=vr0=vr0dc8meaabaqaciaacaGaaeqabaWaaeGaeaaakeaaimaacqWFse=uaaa@3845@. ROC curve for learned structure S
 MathType@MTEF@5@5@+=feaafiart1ev1aaatCvAUfKttLearuWrP9MDH5MBPbIqV92AaeXatLxBI9gBamrtHrhAL1wy0L2yHvtyaeHbnfgDOvwBHrxAJfwnaebbnrfifHhDYfgasaacH8akY=wiFfYdH8Gipec8Eeeu0xXdbba9frFj0=OqFfea0dXdd9vqai=hGuQ8kuc9pgc9s8qqaq=dirpe0xb9q8qiLsFr0=vr0=vr0dc8meaabaqaciaacaGaaeqabaWaaeGaeaaakeaaimaacqWFse=uaaa@3845@ trained on lac repressor, tested on lysozyme. The blue line is the ROC curve. The red line is the convex hull of the ROC curve. The circled point which lies on both of these curves is the classifier with the selected threshold (cost ratio = 3.0).

Structure S
 MathType@MTEF@5@5@+=feaafiart1ev1aaatCvAUfKttLearuWrP9MDH5MBPbIqV92AaeXatLxBI9gBamrtHrhAL1wy0L2yHvtyaeHbnfgDOvwBHrxAJfwnaebbnrfifHhDYfgasaacH8akY=wiFfYdH8Gipec8Eeeu0xXdbba9frFj0=OqFfea0dXdd9vqai=hGuQ8kuc9pgc9s8qqaq=dirpe0xb9q8qiLsFr0=vr0=vr0dc8meaabaqaciaacaGaaeqabaWaaeGaeaaakeaaimaacqWFse=uaaa@3845@ appeared to perform worse than the naïve structure during heterogeneous cross-validation, especially when trained on lac repressor and tested on lysozyme data. Here, AUC decreased from 0.80 to 0.72 despite lower effect error rates at the selected threshold (0.33 *vs *0.52). The low AUC value of 0.72 may be deceptive since a significant number of points on the ROC curve lie below the convex hull (Figure 3) and as such are non-optimal classifiers [32]. Therefore, measuring the performance of a classifier which represents a single point on both the ROC curve and the convex hull (circled in Figure 3) was more useful in this case. As described in Methods, we chose the point at cost ratio 3.0 (where false positives cost three times more than false negatives) as this helps balance the 'effect' and 'no effect' misclassification error rates (important in datasets such as ours that are biased towards negative examples). At this selected threshold, overall error (0.17) and effect error rate (0.33) were lower for structure S
 MathType@MTEF@5@5@+=feaafiart1ev1aaatCvAUfKttLearuWrP9MDH5MBPbIqV92AaeXatLxBI9gBamrtHrhAL1wy0L2yHvtyaeHbnfgDOvwBHrxAJfwnaebbnrfifHhDYfgasaacH8akY=wiFfYdH8Gipec8Eeeu0xXdbba9frFj0=OqFfea0dXdd9vqai=hGuQ8kuc9pgc9s8qqaq=dirpe0xb9q8qiLsFr0=vr0=vr0dc8meaabaqaciaacaGaaeqabaWaaeGaeaaakeaaimaacqWFse=uaaa@3845@ than the naïve structure (0.20 and 0.52 respectively). However, MCC value was also lower (0.30 *vs *0.40) and no effect error rate was higher (0.16 *vs *0.10) which illustrates the difficulty in selecting a measure to compare different models not only between different studies but within the same study as well.

#### Missing structural information (all:noS and noS:noS)

The model learned from all the variables and tested using only evolutionary information (*all*:*noS*, Table 3, column 2) performed poorly achieving AUC values less than 0.50 (worse than random) and error rates above 0.75. Given the number of connections in S
 MathType@MTEF@5@5@+=feaafiart1ev1aaatCvAUfKttLearuWrP9MDH5MBPbIqV92AaeXatLxBI9gBamrtHrhAL1wy0L2yHvtyaeHbnfgDOvwBHrxAJfwnaebbnrfifHhDYfgasaacH8akY=wiFfYdH8Gipec8Eeeu0xXdbba9frFj0=OqFfea0dXdd9vqai=hGuQ8kuc9pgc9s8qqaq=dirpe0xb9q8qiLsFr0=vr0=vr0dc8meaabaqaciaacaGaaeqabaWaaeGaeaaakeaaimaacqWFse=uaaa@3845@ and the potential for inferring the missing structural information in the test data, the *all*:*noS *model was surprisingly worse than the *noS*:*noS *model (Table 3, column 3).

There could be a number of reasons for the poor performance of the *all*:*noS *model. The model may have learned during training that structural information is more important to prediction than evolutionary information. Consequently, without structural information during testing, the model has problems since it has down-weighted the evolutionary nodes relative to the structural nodes. Alternatively, it may not be possible to accurately infer values at the structural nodes from evolutionary information. By contrast, it is essential that the *noS*:*noS *model makes full use of the evolutionary information since structural information is unavailable in both training and testing. Even though cross validation results with *noS*:*noS *were worse than the *all*:*all *model with AUC values ranging from 0.65 – 0.73 and overall error rates up to 0.27, they were better than the *all*:*noS *since full weight is given to the evolutionary nodes.

#### Missing evolutionary information (all:noE and noE:noE)

When marginalising over unknown evolutionary variables (*all*:*noE*, Table 3, column 4), predictions in most cases were comparable to the *all*:*all *model. However, poor results were achieved during homogeneous cross validation on mixed data and heterogeneous cross validation, especially training on lysozyme and testing on lac repressor data (AUC 0.58). In these cases, it appears that values at the evolutionary nodes with missing data could not be predicted accurately from the structural information during testing thus confusing the model. As expected, the *noE*:*noE *model trained and tested using structural variables only performed as well as the all:all model across all cross validation tests (Table 3, column 5).

### Tolerance to incomplete training data

**Figure 4:**
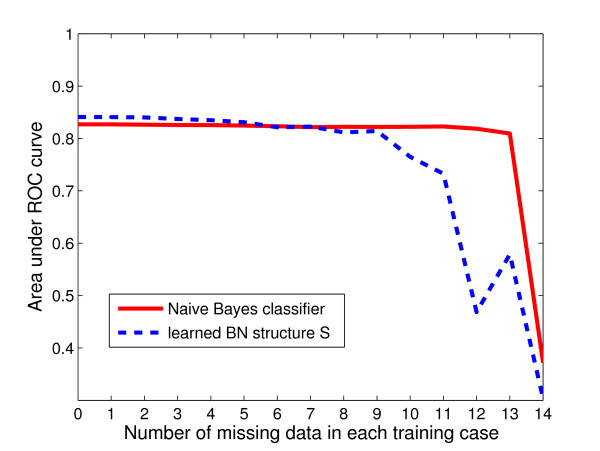
**Classifier performance**. Performance of naïve Bayes classifier and structure S
 MathType@MTEF@5@5@+=feaafiart1ev1aaatCvAUfKttLearuWrP9MDH5MBPbIqV92AaeXatLxBI9gBamrtHrhAL1wy0L2yHvtyaeHbnfgDOvwBHrxAJfwnaebbnrfifHhDYfgasaacH8akY=wiFfYdH8Gipec8Eeeu0xXdbba9frFj0=OqFfea0dXdd9vqai=hGuQ8kuc9pgc9s8qqaq=dirpe0xb9q8qiLsFr0=vr0=vr0dc8meaabaqaciaacaGaaeqabaWaaeGaeaaakeaaimaacqWFse=uaaa@3845@ with parameters learned from incomplete data. The AUC (area under the ROC curve) is plotted against the number of nodes (n) randomly chosen to have missing data within the test examples.

Bayesian networks are capable of learning model parameters from incomplete data. Here we test the tolerance of the Bayesian networks by training on incomplete data. In every training example, we hide *n *nodes (chosen randomly for each training case). We do this for the naïve Bayes classifier, and the learned structure S
 MathType@MTEF@5@5@+=feaafiart1ev1aaatCvAUfKttLearuWrP9MDH5MBPbIqV92AaeXatLxBI9gBamrtHrhAL1wy0L2yHvtyaeHbnfgDOvwBHrxAJfwnaebbnrfifHhDYfgasaacH8akY=wiFfYdH8Gipec8Eeeu0xXdbba9frFj0=OqFfea0dXdd9vqai=hGuQ8kuc9pgc9s8qqaq=dirpe0xb9q8qiLsFr0=vr0=vr0dc8meaabaqaciaacaGaaeqabaWaaeGaeaaakeaaimaacqWFse=uaaa@3845@, and vary *n *from 0 to 14. The CPTs are learned using the iterative EM algorithm on the missing values. Figure 4 shows the results of homogeneous cross-validation when trained on incomplete data from the 'mixed' dataset, and tested when all nodes are observed. Note that using this method, different sets of *n *nodes are chosen to have missing data between different training cases, therefore here we were testing the general ability of the Bayesian network to tolerate incomplete data rather than the effect of when certain nodes were missing data in all examples (as in the previous section).

Figure 4 shows that the performance of both the naïve and S
 MathType@MTEF@5@5@+=feaafiart1ev1aaatCvAUfKttLearuWrP9MDH5MBPbIqV92AaeXatLxBI9gBamrtHrhAL1wy0L2yHvtyaeHbnfgDOvwBHrxAJfwnaebbnrfifHhDYfgasaacH8akY=wiFfYdH8Gipec8Eeeu0xXdbba9frFj0=OqFfea0dXdd9vqai=hGuQ8kuc9pgc9s8qqaq=dirpe0xb9q8qiLsFr0=vr0=vr0dc8meaabaqaciaacaGaaeqabaWaaeGaeaaakeaaimaacqWFse=uaaa@3845@ structures (measured by AUC value) were robust to incomplete training data, with an area under the ROC curve of over 0.80 maintained even when nine of the fifteen nodes were not observed in every example. With very sparse data (more than 9 nodes hidden), the naïve Bayes classifier performed better than the learned structure. This was probably because the conditional probability tables (CPTs) of the naïve structure only model the relationship of *effect* with each variable, whereas the CPTs of S
 MathType@MTEF@5@5@+=feaafiart1ev1aaatCvAUfKttLearuWrP9MDH5MBPbIqV92AaeXatLxBI9gBamrtHrhAL1wy0L2yHvtyaeHbnfgDOvwBHrxAJfwnaebbnrfifHhDYfgasaacH8akY=wiFfYdH8Gipec8Eeeu0xXdbba9frFj0=OqFfea0dXdd9vqai=hGuQ8kuc9pgc9s8qqaq=dirpe0xb9q8qiLsFr0=vr0=vr0dc8meaabaqaciaacaGaaeqabaWaaeGaeaaakeaaimaacqWFse=uaaa@3845@ depend on the relationship between multiple variables. From Figure 2, we can see that a number of nodes are dependent on three variables in S
 MathType@MTEF@5@5@+=feaafiart1ev1aaatCvAUfKttLearuWrP9MDH5MBPbIqV92AaeXatLxBI9gBamrtHrhAL1wy0L2yHvtyaeHbnfgDOvwBHrxAJfwnaebbnrfifHhDYfgasaacH8akY=wiFfYdH8Gipec8Eeeu0xXdbba9frFj0=OqFfea0dXdd9vqai=hGuQ8kuc9pgc9s8qqaq=dirpe0xb9q8qiLsFr0=vr0=vr0dc8meaabaqaciaacaGaaeqabaWaaeGaeaaakeaaimaacqWFse=uaaa@3845@, which perhaps explains the performance decrease when the model is not trained on sets of four or more variables. For example, when 11 variables are missing, an AUC value of 0.73 is achieved by S
 MathType@MTEF@5@5@+=feaafiart1ev1aaatCvAUfKttLearuWrP9MDH5MBPbIqV92AaeXatLxBI9gBamrtHrhAL1wy0L2yHvtyaeHbnfgDOvwBHrxAJfwnaebbnrfifHhDYfgasaacH8akY=wiFfYdH8Gipec8Eeeu0xXdbba9frFj0=OqFfea0dXdd9vqai=hGuQ8kuc9pgc9s8qqaq=dirpe0xb9q8qiLsFr0=vr0=vr0dc8meaabaqaciaacaGaaeqabaWaaeGaeaaakeaaimaacqWFse=uaaa@3845@, whereas when 12 variables are missing, performance decreases to that of random classifier (AUC < 0.5). Nevertheless, overall tolerance to incomplete training data by both Bayesian networks was encouraging considering the potential sparsity of either evolutionary and structural information for a significant number of proteins, especially structural genomics targets. Other machine learning methods such as SVMs or decision trees are unable to handle incomplete data in this way.

### Training set size

**Figure 5:**
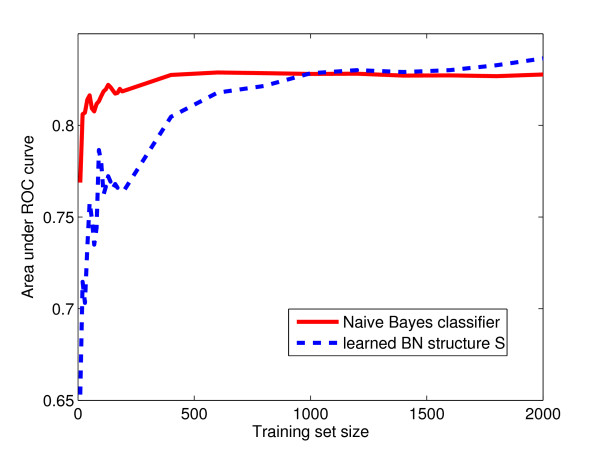
**Training set size**. Performance of naïve Bayes classifier and structure S
 MathType@MTEF@5@5@+=feaafiart1ev1aaatCvAUfKttLearuWrP9MDH5MBPbIqV92AaeXatLxBI9gBamrtHrhAL1wy0L2yHvtyaeHbnfgDOvwBHrxAJfwnaebbnrfifHhDYfgasaacH8akY=wiFfYdH8Gipec8Eeeu0xXdbba9frFj0=OqFfea0dXdd9vqai=hGuQ8kuc9pgc9s8qqaq=dirpe0xb9q8qiLsFr0=vr0=vr0dc8meaabaqaciaacaGaaeqabaWaaeGaeaaakeaaimaacqWFse=uaaa@3845@ with parameters learned sequentially. The AUC (area under the ROC curve) is plotted against the number of training examples.

In order to assess how much data is needed for training the Bayesian networks, sequential learning of the model parameters was performed. The 'mixed' dataset was divided into two. One half was used as the test validation set, and the Bayesian networks were trained on the other half. Figure 5 shows a plot of training set size vs. classifier performance, measured using area under the ROC curve. The result is as expected. The naïve model (with its 43 parameters) gradually improves its performance as its parameters are sequentially learned, with excellent performance after 400 examples (and good after as few as 50). The learned BN structure S
 MathType@MTEF@5@5@+=feaafiart1ev1aaatCvAUfKttLearuWrP9MDH5MBPbIqV92AaeXatLxBI9gBamrtHrhAL1wy0L2yHvtyaeHbnfgDOvwBHrxAJfwnaebbnrfifHhDYfgasaacH8akY=wiFfYdH8Gipec8Eeeu0xXdbba9frFj0=OqFfea0dXdd9vqai=hGuQ8kuc9pgc9s8qqaq=dirpe0xb9q8qiLsFr0=vr0=vr0dc8meaabaqaciaacaGaaeqabaWaaeGaeaaakeaaimaacqWFse=uaaa@3845@ has 182 free parameters and it out performs the naïve classifier after 1000 training examples.

### Interpreting the structures

**Figure 6:**
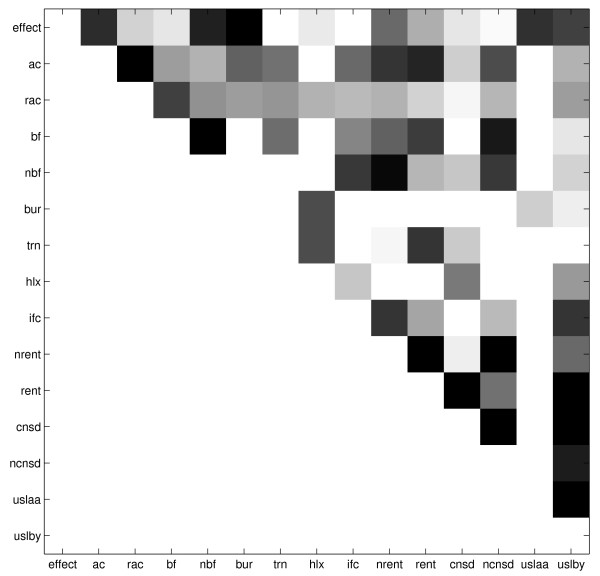
**Posterior distribution of relationships**. Strength of relationships between variables, identified through analysis of edges connecting pairs of nodes in MCMC structure learning. A dark square indicates a strong relationship; a white square a weak relationship.

The learned structure S
 MathType@MTEF@5@5@+=feaafiart1ev1aaatCvAUfKttLearuWrP9MDH5MBPbIqV92AaeXatLxBI9gBamrtHrhAL1wy0L2yHvtyaeHbnfgDOvwBHrxAJfwnaebbnrfifHhDYfgasaacH8akY=wiFfYdH8Gipec8Eeeu0xXdbba9frFj0=OqFfea0dXdd9vqai=hGuQ8kuc9pgc9s8qqaq=dirpe0xb9q8qiLsFr0=vr0=vr0dc8meaabaqaciaacaGaaeqabaWaaeGaeaaakeaaimaacqWFse=uaaa@3845@ is one of many Markov equivalent structures which could have been learned from this data. There are also many other network structures which could suitably encode the relationships between the variables. Using Markov chain Monte Carlo (MCMC) methods, we constructed a set of 'good' model structures, and averaged over these models to form a posterior distribution of model structures. Figure 6 shows a plot of the frequency of connections made in the set of 'good' structures from ten runs of the MCMC simulation over 10000 samples, after a 'burnin' of 1000 samples. The darker squares indicate a higher observed frequency of an edge connecting each pair of nodes. From this, one can identify strong relationships between highly correlated variables.

The use of MCMC methods to study the posterior distribution over networks has the advantage of revealing relationships between the input variables. For instance, in Figure 6, the top row shows which variables are most strongly related to *effect*, and this is used later to develop simplified classifiers.

However, biologically meaningful relationships between the other variables are also revealed. With the exception of the trivial relationship between *ac* and *rac*, the second row of Figure 6 shows strong links between *ac*, *nrent*, and *rent*, reflecting a well known biochemical relationship between solvent accessibility of residues and phylogenetic variability: the solvent exposed surface loops of protein structures show greater evolutionary variability than the unexposed hydrophobic core residues. Similar effects that concur with known protein chemistry relate measures of flexibility (*nbf*, *bf*) to phylogenetic variability. Equally understandable are the strong link between G and P residues in turns (*trn*) and evolutionary conservation at the specific sequence position of G/P (indicated by *rent*, *cnsd*) but not to a neighbourhood measure (*nrent*, *ncnsd*), and the relationship between protein interface positions (*ifc*) and neighbourhood flexibility measures (*nbf*).

From Figure 6, one can see that the *effect* node has the strongest relationships with *bur*, *nbf*, *ac*, *uslaa*, and *uslby* (in descending order). There are very few direct connections between *effect* and *trn*, *hlx*, *ifc*, *cnsd*, and *ncnsd*. As expected, nodes such as *bf* and *nbf*, and *rent* and *nrent* are highly correlated, which suggests some redundancy within the network and one node could be used to predict the value of the other. Both structural and evolutionary information are represented by the nodes most frequently directly connected to *effect*, although the top three most common nodes, *bur*, *nbf* and *ac*, represent only structural information. This, together with the strong performance of the Bayes classifier without evolutionary information (Table 2, columns 4 and 5), suggests that evolutionary properties of the mutated residue have little direct influence on prediction if structural information is present.

Our finding that solvent accessible area of the native amino acid, whether the amino acid is charged at a buried site, and the flexibility of its structural neighbourhood are all important predictors of effect agrees to some extent with Chasman and Adams (2001) who found that structure based accessibility and B-factor features have the most discriminatory power. The strong performance of accessibility measures probably reflects the finding of [5] and [6] that mutations affecting the hydrophobic core are more likely to destabilise the protein and thus affect function. Perhaps mutations on the surface are more likely affect function if they are conserved, as suggested by the strong relationship between accessibility and phylogenetic entropy (*ac* with *rent* and *nrent*). Conversely, whether or not the mutation breaks either a helix or turn does not appear to be critical to predicting effect although, again, secondary structure information may become more powerful when used in conjunction with other features.

### A simplified Bayesian network

Whilst the nodes directly connected to the *effect* node are not essential to prediction if certain other nodes are present (as demonstrated by the removal of the structural flexibility nodes *nbf* and *bf*, with no significant loss of performance), in theory, the value of the *effect* node can be predicted using only the nodes which are directly connected to it in the learned structures. The other variables become d-separated from *effect*; i.e. with a structure, and the conditional independence relations it implies, the effect node is conditionally dependent on only the nodes it is connected to when they are observed.

**Figure 7:**
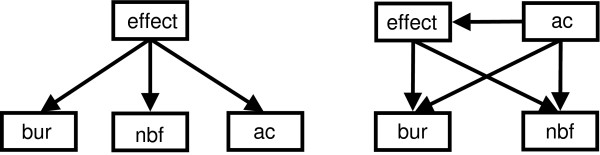
**Simplified Bayesian networks**. Four node networks using the three key variables shown to have the strongest relationship with the effect. (a) Naïve Bayes classifier, (b) learned Bayesian network structure S
 MathType@MTEF@5@5@+=feaafiart1ev1aaatCvAUfKttLearuWrP9MDH5MBPbIqV92AaeXatLxBI9gBamrtHrhAL1wy0L2yHvtyaeHbnfgDOvwBHrxAJfwnaebbnrfifHhDYfgasaacH8akY=wiFfYdH8Gipec8Eeeu0xXdbba9frFj0=OqFfea0dXdd9vqai=hGuQ8kuc9pgc9s8qqaq=dirpe0xb9q8qiLsFr0=vr0=vr0dc8meaabaqaciaacaGaaeqabaWaaeGaeaaakeaaimaacqWFse=uaaa@3845@_3_.

We tested this hypothesis by constructing two simple four node networks: a naïve structure (Figure 7a), and structure S
 MathType@MTEF@5@5@+=feaafiart1ev1aaatCvAUfKttLearuWrP9MDH5MBPbIqV92AaeXatLxBI9gBamrtHrhAL1wy0L2yHvtyaeHbnfgDOvwBHrxAJfwnaebbnrfifHhDYfgasaacH8akY=wiFfYdH8Gipec8Eeeu0xXdbba9frFj0=OqFfea0dXdd9vqai=hGuQ8kuc9pgc9s8qqaq=dirpe0xb9q8qiLsFr0=vr0=vr0dc8meaabaqaciaacaGaaeqabaWaaeGaeaaakeaaimaacqWFse=uaaa@3845@_3 _(Figure 7b) learned using the greedy search algorithm and the BIC scoring function as above. These networks consisted of only the three nodes, *bur*, *nbf* and *ac*, with the strongest relationships with *effect* as shown in Figure 6.

Across all cross-validation tests, the four node naïve Bayes classifier trained and tested using only the three key variables (*key*:*key*, Table 2, final column) performed extremely well with only a minor decrease in performance over the *all*:*all *model. In homogeneous cross validation, AUC values ranged from 0.77 to 0.80 and the maximum overall error rate was just 0.21. In heterogeneous cross validation tests, the AUC also remained high (0.77 and 0.79) with overall error rates as low as 0.16 for training on lac repressor and testing on lysozyme data. There were no significant differences between the performance of the four node learned structure (*key*:*key*, Table 3, final column) and that of the naïve structure, which suggests little value in the connections between variables.

## Conclusion

We have applied Bayesian networks to the task of predicting whether or not missense mutations will affect protein function with comparable performance to other machine learning methods. However, the strength of the Bayesian network lies in its ability to handle incomplete data and to encode relationships between variables; both of which were exploited here to derive some biological insight into how a missense mutation affects protein function.

A number of models were learned in this work. Due to the unbalanced datasets we analysed ROC curves and selected a suitable cost ratio in order to choose a probability threshold for the classifiers. This allowed us to compare classifiers in a meaningful way. From this analysis we concluded that a naïve network structure is sufficient for accurate prediction of the effect of a missense mutation with AUC values around 0.80. We also found that the structural environment of the amino acid is a far better predictor of the functional consequences of a missense mutation than phylogenetic information. This was demonstrated by the more accurate performance of a naïve classifier that just uses structural information compared to that which uses just evolutionary information. There were no significant performance gains when using a learned network structure, however the learned structure did allow relationships between variables to be analysed, in particular by analysing the posterior distribution of model structures, we found the top three strongest connections with the effect node all involved structural nodes. With this in mind, we derived a simplified Bayesian network that used just these three structural descriptors (solvent accessible area of the native amino acid, whether the amino acid is charged at a buried site, and the flexibility of its structural neighbourhood) without significant decrease in performance. Given the importance of structure, it would be interesting to learn if certain amino acid changes are more predictive of effect than others. For example, both cysteine, which forms disulphide bridges, and proline, with its unique ring structure, are often critical to the integrity of a protein structure so one would expect a mutation involving either of these residues to change the structure significantly. This will provide the basis for future work.

## Methods

### Evaluation measures

A number of measures were applied to evaluate each classifier: error rates (fraction of mis-classified examples), sensitivity (true positive rate) and specificity (true negative rate). We also used Matthews' correlation coefficient (MCC), which is a correlation measure designed for comparison of unbalanced datasets such as ours. A value of +1 indicates perfect classification, and -1 indicates misclassification of every example. The MCC is defined as:

MCC=(TP×TN)−(FP×FN)(TP+FP)(TP+FN)(TN+FP)(TN+FN)
 MathType@MTEF@5@5@+=feaafiart1ev1aaatCvAUfKttLearuWrP9MDH5MBPbIqV92AaeXatLxBI9gBaebbnrfifHhDYfgasaacH8akY=wiFfYdH8Gipec8Eeeu0xXdbba9frFj0=OqFfea0dXdd9vqai=hGuQ8kuc9pgc9s8qqaq=dirpe0xb9q8qiLsFr0=vr0=vr0dc8meaabaqaciaacaGaaeqabaqabeGadaaakeaacqWGnbqtcqWGdbWqcqWGdbWqcqGH9aqpdaWcaaqaaiabcIcaOiabdsfaujabdcfaqjabgEna0kabdsfaujabd6eaojabcMcaPiabgkHiTiabcIcaOiabdAeagjabdcfaqjabgEna0kabdAeagjabd6eaojabcMcaPaqaamaakaaabaGaeiikaGIaemivaqLaemiuaaLaey4kaSIaemOrayKaemiuaaLaeiykaKIaeiikaGIaemivaqLaemiuaaLaey4kaSIaemOrayKaemOta4KaeiykaKIaeiikaGIaemivaqLaemOta4Kaey4kaSIaemOrayKaemiuaaLaeiykaKIaeiikaGIaemivaqLaemOta4Kaey4kaSIaemOrayKaemOta4KaeiykaKcaleqaaaaaaaa@5F65@

where TP are true positives, TN are true negatives, FP are false positives, and FN are false negatives obtained from evaluating the classifier on the test data.

Since we have a Bayesian network classifier, with a probability associated with each classification, the metrics above depend on the value of the classification threshold *p *that is used. To assess performance across a range of values of the probability threshold we plotted a receiver operator characteristic (ROC) curve. The ROC curve is a plot of the sensitivity versus (1-specificity) for all feasible ratios of costs associated with misclassification errors (equivalent to plotting true positive rate versus false positive rate). The area under the curve (AUC) is a measure of the performance of a binary classifier. A classifier no better than random gives an AUC of 0.5, a perfect classifier gives an AUC of 1.0.

### Choosing a classification threshold

In order to perform a fair comparison of classifiers, we choose the classification threshold *p*, represented by a point on the ROC curve for which the curve has a gradient (ΔTPrate/ΔFPrate) of *C*_*FP*_/*C*_*FN *_– the ratio of costs between False Positives and False Negatives, and which is closest to the point (0, 1). In this work we use a cost ratio of 3.0, due to the unbalanced nature of the datasets containing 3742 mutations which have no effect on protein function and 1135 which do effect protein function. This is close to 3:1, and by weighting the cost of a false positive, *C*_*FP*_, as three times more costly (to the classifier) than a false negative, *C*_*FN*_, we obtain a classifier with reasonably well balanced error rates. This means the classifier is less likely to predict everything as an effect (than with an equal cost ratio of 1.0) and make many false positive errors, which would give a high effect error rate. (Without doing this, we may be comparing classifiers with very different properties. i.e. ones with quite different specificities and sensitivities). The method is applied to the ROC curves obtained from the probabilistic classification scheme and we present the results for the classification threshold *p *corresponding to the point on the convex hull of the ROC curve where the gradient is closest 3.0. (We choose a point on the convex hull since any point on the ROC curve not on the convex hull is a non-optimal classifier [32]).

### Data discretization

There were a number of challenges buried in these data. Continuous data was non-Gaussian, making it unsuitable for modelling as a continuous Gaussian node in a Bayesian network. There were also no obvious boundaries at which to separate the data into discrete categories. Our solution was to fit a number of Gaussians to the data using an Expectation-Maximisation based algorithm that automatically chooses the number of classes. It begins with one Gaussian, and iteratively splits the Gaussian with the largest weight, until adding extra classes does not increase the maximum likelihood of the model. Full details are provided below. This allowed us to form discrete classes from continuous data, which gave better performance than simply splitting the data into three classes of equal range (results not shown). We therefore used this strategy in all our analyses.

#### The E-M algorithm

The Expectation Maximisation algorithm is a well established efficient algorithm for fitting Gaussian mixture models to data. The main draw-back of the algorithm is its sensitivity to initialisation, and the need for multiple runs with different numbers of mixtures in order to choose the maximum likelihood model. Here we present a adaptation to the method which is deterministic and automatically chooses the number of Gaussians. It begins with one Gaussian, and iteratively splits the Gaussian with the largest weight, until adding extra mixtures does not increase the maximum likelihood of the model. Given a data set **X **= {x_1_,..., x_*N*_} of *N *cases of *d*-dimensional data, a single cluster ***μ***_1 _is initialised at the mean of the data. A Gaussian with diagonal covariance equal to the standard deviation of the data in each dimension is placed at ***μ***_1 _The weight of this cluster is set to one *p*(*j *= 1) = 1.

μ1=1N∑i=1Nxis1=1N∑i=1N(xi−μ1)(xi−μ1)Tp(j=1)=1
 MathType@MTEF@5@5@+=feaafiart1ev1aaatCvAUfKttLearuWrP9MDH5MBPbIqV92AaeXatLxBI9gBaebbnrfifHhDYfgasaacH8akY=wiFfYdH8Gipec8Eeeu0xXdbba9frFj0=OqFfea0dXdd9vqai=hGuQ8kuc9pgc9s8qqaq=dirpe0xb9q8qiLsFr0=vr0=vr0dc8meaabaqaciaacaGaaeqabaqabeGadaaakeaafaqabeWabaaabaaccmGae8hVd02aaSbaaSqaaiabigdaXaqabaGccqGH9aqpdaWcaaqaaiabigdaXaqaaiabd6eaobaadaaeWaqaaiabbIha4naaBaaaleaacqWGPbqAaeqaaaqaaiabdMgaPjabg2da9iabigdaXaqaaiabd6eaobqdcqGHris5aaGcbaGaee4Cam3aaSbaaSqaaiabigdaXaqabaGccqGH9aqpdaWcaaqaaiabigdaXaqaaiabd6eaobaadaaeWaqaaiabcIcaOiabbIha4naaBaaaleaacqWGPbqAaeqaaOGaeyOeI0Iae8hVd02aaSbaaSqaaiabigdaXaqabaGccqGGPaqkcqGGOaakcqqG4baEdaWgaaWcbaGaemyAaKgabeaakiabgkHiTiab=X7aTnaaBaaaleaacqaIXaqmaeqaaOGaeiykaKYaaWbaaSqabeaacqWGubavaaaabaGaemyAaKMaeyypa0JaeGymaedabaGaemOta4eaniabggHiLdaakeaacqWGWbaCcqGGOaakcqWGQbGAcqGH9aqpcqaIXaqmcqGGPaqkcqGH9aqpcqaIXaqmaaaaaa@6332@

The probability *p*(*j*|x_*i*_) is calculated for each data point x_*i*_.

For a set of *j *mixtures, the update equations follow. These are iteratively performed until the maximum likelihood is reached, i.e. *ML *= log∑_*i*_∑_*j*_*p*(*j*|x_*i*_)

E-step:

p(j|xi)=p(j)p(xi|j)p(xi)p(xi|j)=1(2π)d2|sj|12exp⁡(−12(xi−μj)sj−1(xi−μj)T)p(j)=∑i=1Np(j|xi)
 MathType@MTEF@5@5@+=feaafiart1ev1aaatCvAUfKttLearuWrP9MDH5MBPbIqV92AaeXatLxBI9gBaebbnrfifHhDYfgasaacH8akY=wiFfYdH8Gipec8Eeeu0xXdbba9frFj0=OqFfea0dXdd9vqai=hGuQ8kuc9pgc9s8qqaq=dirpe0xb9q8qiLsFr0=vr0=vr0dc8meaabaqaciaacaGaaeqabaqabeGadaaakeaafaqaaeWadaaabaGaemiCaaNaeiikaGIaemOAaOMaeiiFaWNaeeiEaG3aaSbaaSqaaiabdMgaPbqabaGccqGGPaqkaeaacqGH9aqpaeGafaGs5dGQ5dG85dGt+dGg=paalaaabaGaemiCaaNaeiikaGIaemOAaOMaeiykaKIaemiCaaNaeiikaGIaeeiEaG3aaSbaaSqaaiabdMgaPbqabaGccqGG8baFcqWGQbGAcqGGPaqkaeaacqWGWbaCcqGGOaakcqWG4baEdaWgaaWcbaGaemyAaKgabeaakiabcMcaPaaaaeaacqWGWbaCcqGGOaakcqqG4baEdaWgaaWcbaGaemyAaKgabeaakiabcYha8jabdQgaQjabcMcaPaqaaiabg2da9aqaamaalaaabaGaeGymaedabaGaeiikaGIaeGOmaidcciGae8hWdaNaeiykaKYaaWbaaSqabeaadaWcaaqaaiabdsgaKbqaaiabikdaYaaaaaGcdaabdiqaaiabbohaZnaaBaaaleaacqWGQbGAaeqaaaGccaGLhWUaayjcSdWaaWbaaSqabeaadaWcaaqaaiabigdaXaqaaiabikdaYaaaaaaaaOGagiyzauMaeiiEaGNaeiiCaa3aaeWaceaacqGHsisldaWcaaqaaiabigdaXaqaaiabikdaYaaacqGGOaakcqqG4baEdaWgaaWcbaGaemyAaKgabeaakiabgkHiTGGadiab+X7aTnaaBaaaleaacqWGQbGAaeqaaOGaeiykaKIaee4Cam3aa0baaSqaaiabdQgaQbqaaiabgkHiTiabigdaXaaakiabcIcaOiabbIha4naaBaaaleaacqWGPbqAaeqaaOGaeyOeI0Iae4hVd02aaSbaaSqaaiabdQgaQbqabaGccqGGPaqkdaahaaWcbeqaaiabdsfaubaaaOGaayjkaiaawMcaaaqaceaadiGaaCzcaiabdchaWjabcIcaOiabdQgaQjabcMcaPaqaaiabg2da9aqaamaaqadabaGaemiCaaNaeiikaGIaemOAaOMaeiiFaWNaeeiEaG3aaSbaaSqaaiabdMgaPbqabaGccqGGPaqkaSqaaiabdMgaPjabg2da9iabigdaXaqaaiabd6eaobqdcqGHris5aaaaaaa@A25C@

M-step:

μj=1p(j)∑i=1Np(j|xi)xisj=1p(j)∑i=1Np(j|xi)(xi−μj)(xi−μj)T
 MathType@MTEF@5@5@+=feaafiart1ev1aaatCvAUfKttLearuWrP9MDH5MBPbIqV92AaeXatLxBI9gBaebbnrfifHhDYfgasaacH8akY=wiFfYdH8Gipec8Eeeu0xXdbba9frFj0=OqFfea0dXdd9vqai=hGuQ8kuc9pgc9s8qqaq=dirpe0xb9q8qiLsFr0=vr0=vr0dc8meaabaqaciaacaGaaeqabaqabeGadaaakeaafaqadeGadaaabaaccmGae8hVd02aaSbaaSqaaiabdQgaQbqabaaakeaacqGH9aqpaeaadaWcaaqaaiabigdaXaqaaiabdchaWjabcIcaOiabdQgaQjabcMcaPaaadaaeWaqaaiabdchaWjabcIcaOiabdQgaQjabcYha8jabbIha4naaBaaaleaacqWGPbqAaeqaaOGaeiykaKIaeeiEaG3aaSbaaSqaaiabdMgaPbqabaaabaGaemyAaKMaeyypa0JaeGymaedabaGaemOta4eaniabggHiLdaakeGabaajaiaaxMaacqqGZbWCdaWgaaWcbaGaemOAaOgabeaaaOqaaiabg2da9aqaamaalaaabaGaeGymaedabaGaemiCaaNaeiikaGIaemOAaOMaeiykaKcaamaaqadabaGaemiCaaNaeiikaGIaemOAaOMaeiiFaWNaeeiEaG3aaSbaaSqaaiabdMgaPbqabaGccqGGPaqkcqGGOaakcqqG4baEdaWgaaWcbaGaemyAaKgabeaakiabgkHiTiab=X7aTnaaBaaaleaacqWGQbGAaeqaaOGaeiykaKIaeiikaGIaeeiEaG3aaSbaaSqaaiabdMgaPbqabaGccqGHsislcqaH8oqBdaWgaaWcbaGaemOAaOgabeaakiabcMcaPmaaCaaaleqabaGaemivaqfaaaqaaiabdMgaPjabg2da9iabigdaXaqaaiabd6eaobqdcqGHris5aaaaaaa@75F2@

When the ML stops increasing, the Gaussian with the largest weight *p*(*j*) described by ***μ***_*j *_and s_*j *_is split into two Gaussians at μj+
 MathType@MTEF@5@5@+=feaafiart1ev1aaatCvAUfKttLearuWrP9MDH5MBPbIqV92AaeXatLxBI9gBaebbnrfifHhDYfgasaacH8akY=wiFfYdH8Gipec8Eeeu0xXdbba9frFj0=OqFfea0dXdd9vqai=hGuQ8kuc9pgc9s8qqaq=dirpe0xb9q8qiLsFr0=vr0=vr0dc8meaabaqaciaacaGaaeqabaqabeGadaaakeaaiiGacqWF8oqBdaqhaaWcbaGaemOAaOgabaGaey4kaScaaaaa@30D5@ and μj−
 MathType@MTEF@5@5@+=feaafiart1ev1aaatCvAUfKttLearuWrP9MDH5MBPbIqV92AaeXatLxBI9gBaebbnrfifHhDYfgasaacH8akY=wiFfYdH8Gipec8Eeeu0xXdbba9frFj0=OqFfea0dXdd9vqai=hGuQ8kuc9pgc9s8qqaq=dirpe0xb9q8qiLsFr0=vr0=vr0dc8meaabaqaciaacaGaaeqabaqabeGadaaakeaaiiGacqWF8oqBdaqhaaWcbaGaemOAaOgabaGaeyOeI0caaaaa@30E0@, each with the same covariance s_*j*_. The new Gaussians are placed +/- a distance of the largest eigenvalue in the direction of the principle eigenvector of the covariance matrix s_*j *_from *μ*_*j*_. (The Gaussians are then renamed). The EM steps above are performed until the maximum likelihood configuration is reached. This process is repeated until the ML score is no higher than with one less Gaussian. At each stage, the centres and covariances of the Gaussians are saved. Thus the algorithm terminates with a set of Gaussians that are at best no better than the set at the previous stage with one less Gaussian, so the saved set from the previous stage is used.

Classification of each data point x_*i *_is taken as a hard classification into the most likely class given by arg_*j *_max *p*(*j*|x_*i*_).

## Authors' contributions

CJN carried out the study, CAM assisted with data sets and result interpretation, JRB advised on biological aspects and result interpretation, AJB and DRW, suggested the study and assisted with result interpretation. All authors approved the final manuscript.
